# Headphones or Speakers? An Exploratory Study of Their Effects on Spontaneous Body Movement to Rhythmic Music

**DOI:** 10.3389/fpsyg.2020.00698

**Published:** 2020-04-21

**Authors:** Agata Zelechowska, Victor E. Gonzalez-Sanchez, Bruno Laeng, Alexander Refsum Jensenius

**Affiliations:** ^1^RITMO Centre for Interdisciplinary Studies in Rhythm, Time and Motion, University of Oslo, Oslo, Norway; ^2^Department of Musicology, University of Oslo, Oslo, Norway; ^3^Department of Psychology, University of Oslo, Oslo, Norway

**Keywords:** headphones, speakers, playback method, embodiment, music-induced movement, sensorimotor synchronization, motion capture, electronic dance music

## Abstract

Previous studies have shown that music may lead to spontaneous body movement, even when people try to stand still. But are spontaneous movement responses to music similar if the stimuli are presented using headphones or speakers? This article presents results from an exploratory study in which 35 participants listened to rhythmic stimuli while standing in a neutral position. The six different stimuli were 45 s each and ranged from a simple pulse to excerpts from electronic dance music (EDM). Each participant listened to all the stimuli using both headphones and speakers. An optical motion capture system was used to calculate their quantity of motion, and a set of questionnaires collected data about music preferences, listening habits, and the experimental sessions. The results show that the participants on average moved more when listening through headphones. The headphones condition was also reported as being more tiresome by the participants. Correlations between participants' demographics, listening habits, and self-reported body motion were observed in both listening conditions. We conclude that the playback method impacts the level of body motion observed when people are listening to music. This should be taken into account when designing embodied music cognition studies.

## 1. Introduction

Thinking about music cognition as a process that happens not only in the mind, but also in the body, is becoming increasingly popular in empirical music research (Leman, 2007). This can be seen in a growing amount of research on music-related body movement, both in performance and perception (Gritten and King, 2006, 2011). Many of the existing studies in the field of embodied music cognition have focused on fairly large-scale body movement, such as, people dancing (Toiviainen et al., 2010; Burger et al., 2013) or walking (Styns et al., 2007; Van Dyck et al., 2015). We have been interested in understanding more about how music may induce body movement also when people try not to move. Using a “standstill” paradigm, we have shown that music may lead to spontaneous body movement, albeit at a very small scale (Jensenius et al., 2017; González Sánchez et al., 2018; González Sánchez et al., 2019). These studies have been done using loudspeakers as the playback method of the music stimuli. Given the small spatial range of the movements we are investigating—most people's head movement is on average around 7 mm/s when standing still—we have asked ourselves whether the playback method has an impact on the result. Past research in speech and music perception has shown that using headphones and speakers can lead to different experimental results. However, as far as we can see there are no studies that have examined the potential impact of such playback technologies on bodily responses to music. Given their distinctive acoustic and psychoacoustic properties, as well as different physical and psychological affordances, one could expect that the use of headphones and speakers would also shape the embodied experience of music. The aim of this article is to explore how headphones and speakers can affect bodily responses to music, and, in particular, spontaneous body movement.

### 1.1. Headphones vs. Speakers

Initially invented as equipment to be used by telephone operators over 100 years ago, headphones have evolved to become one of today's most popular commercial audio products. There are numerous types of headphones available, such as around-ear, over-ear, in-ear, and conductive. These can again be designed in different ways, for example, with open or closed capsules. To simplify the discussion, all of these will be referred to as “headphones” in this article. We acknowledge that various types (and brands) of headphones impact the final sound in different ways, and deserve a more detailed study in itself. This article, however, will focus on the even larger differences between headphones and speakers.

#### 1.1.1. Main Differences Between Headphones and Speakers

Headphones are an important part of the everyday lives of millions of people around the world. They surpass loudspeakers in terms of their portability, privateness, and affordability. Headphones have become the default playback device for those who enjoy listening to music on the move (walking, running, cycling, etc.), and those who share their acoustic environment with others (shared housing, offices, public transport, etc.). In terms of value for money, high-fidelity headphones are usually more affordable than equally good loudspeaker systems. However, even though headphones have grown in popularity, many people prefer to listen to music on speakers, ranging from small portable mono speakers to high-end multichannel sound systems. Speakers are usually listened to from a distance, which better resembles a natural acoustic environment, and this also prevents the “in-head” feeling associated with sound played through headphones (Stankievech, 2007). Listening on speakers brings the sound alive in the space, and eliminates the problem of “splitting” the sound between the left and right ears, as in the case with headphones. Thus, the spatial representation of sound is different if one listens to the same musical recording on speakers or headphones.

One important bodily difference between headphones and speakers is their visceral impact. Speakers enable sound to be perceived as vibrations in the body, and not only in the ear canal. Such physical sensations are crucial to the perception of low frequencies (McMullin, 2017). Low frequencies, in turn, have a strong impact on the human vestibular system (Todd et al., 2008), which is associated with the sensation of body movement (Todd and Lee, 2015). Furthermore, headphones are typically designed in such way that they block the ear canal or cover the ear lobe, which effectively dampens environmental sounds. This can impair user safety, such as when using headphones in traffic, and can potentially affect postural control. The presence of a continuous auditory input is an important factor in maintaining balance (Gandemer et al., 2016). It has been shown that both soundproof environments and wearing ear defenders significantly increase postural sway in healthy subjects (Kanegaonkar et al., 2012). Similar effects might result from covering the ears with headphones; however, to our knowledge, this has not been systematically investigated. At the same time, there is an indication that noise-canceling headphones, which have an active signal processing unit programmed to cancel sounds from the environment, can disrupt balance. A search of Internet reviews and forums shows that users frequently report experiencing headaches, disorientation, nausea, and dizziness when using such headphones. These are only anecdotal evidence, but there is at least one scientific report of a medical case in which noise-canceling headphones had negative consequences on the vestibular system (Dan-Goor and Samra, 2012).

In addition to the psychoacoustic and physiological differences in users' experience of headphones and speakers, there can also be psychological differences. Headphones may be perceived as a less comfortable playback method, since they have to be worn on the body. Some may perceive the proximity of the sound from headphones as invasive, while others may experience the closeness as intimate (Kallinen and Ravaja, 2007) (this may differ not only from person to person, but also depending on the circumstances and type of music). Last, but not least, there are important social differences between the experience of the two playback methods. Headphones create an isolated “bubble,” within which one can listen to music privately. On the contrary, music played over speakers affords a shared experience, whether desired or not. Thus, listening to music on headphones can heighten feelings of introspection, intimacy, or safety (but also isolation). Listening to music on speakers, on the other hand, can lead to heightened social awareness, self-consciousness, and a lack of privacy (but also inclusiveness).

To conclude, the two playback methods have both advantages and disadvantages, and these should be taken into account when designing embodied music cognition experiments.

#### 1.1.2. The Use of Headphones and Speakers in Embodied Music Cognition Studies

To get an overview of how different playback methods are used in embodied music cognition research, we have reviewed some of the experimental studies on body movement to music that were carried out over the past 15 years (Table 1). While the sample is not exhaustive, the selected articles provide an overview of various types of music-related body movement: movement synchronization to music, body sway to music, spontaneous dance, and the experience of groove and the urge to move to music. Contrary to our expectation, most of the reviewed studies used headphones as playback method. This surprised us, since we thought that research on human body movement would use speakers to allow for free movement in space. When it comes to the quality of the equipment used, it ranges from basic consumer products (e.g., Sennheiser HD 62 TV headphones) to professional equipment (e.g., Sennheiser HD60 or AKG 271 MkII headphones). In several of the studies, however, the specific brand and model are not reported, and information on the type of headphones used is also missing. The level of detail in reporting on speaker type and brand is equally varied. Some of the studies use a pair of stereo speakers, some use only one speaker, while others are based on a multi-channel speaker setup. Those that have mentioned the speaker brand use studio quality equipment (most often different types of Genelec speakers), but one article does not report on speaker brand and type.

**Table 1:** An overview of some relevant studies on body movement in response to music.

**References**	**Title**	***N***	**Headphones/Speakers**	**Loudness**
Edworthy and Waring, 2006	The effects of music tempo and loudness level on treadmill exercise	30	Headphones (personal)	2 levels: ~60 and 80 dB
Carrick et al., 2007	Posturographic changes associated with music listening	266	Headphones (earphones)	Not reported
Styns et al., 2007	Walking on music	20	Headphones (Sennheiser HD 62 TV)	Not reported
Forti et al., 2010	The influence of music on static posturography	12	Headphones	Adjusted for participant comfort (range 60–80 dB)
Toiviainen et al., 2010	Embodied meter: Hierarchical eigenmodes in music-induced movement	18	Not reported	Not reported
Van Dyck et al., 2010	The impact of the bass drum on human dance movement	100	Speakers (four Metro MX100, placed in the corners)	Range 70-90 dB depending on point in time, average level not reported
Demos et al., 2012	Rocking to the beat: Effects of music and partner's movements on spontaneous interpersonal coordination	48	Not reported	Not reported
Burger et al., 2013	Influences of rhythm and timbre-related musical features on characteristics of music-induced movement	60	Speakers (two Genelec 8030A)	Not reported
Kilchenmann and Senn, 2015	Microtiming in Swing and Funk affects the body movement behavior of music expert listeners	160	Headphones (AKG 271 MkII)	Playback loudness was adjusted
Pagnacco et al., 2015	Effect of tone-based sound stimulation on balance performance of normal subjects: Preliminary investigation	39	Headphones (high-fidelity)	Adjusted for participant comfort
Van Dyck et al., 2015	Spontaneous entrainment of running cadence to music tempo	16	Headphones (Sennheiser HD60 with Sennheiser HDR130 audio transmitter)	Not reported
Ross et al., 2016	Influence of musical groove on postural sway	40	Headphones (noise-minimizing)	Adjusted for participant comfort
Witek et al., 2017	Syncopation affects free body–movement in musical groove	25	Speakers	75 dB
Burger et al., 2018	Synchronization to metrical levels in music depends on low-frequency spectral components and tempo	30	Speakers (two Genelec 8030A)	Not reported
Coste et al., 2018	Standing or swaying to the beat: Discrete auditory rhythms entrain stance and promote postural coordination stability	20	Headphones (wireless earphones)	Adjusted for participant comfort
Etani et al., 2018	Optimal tempo for groove: Its relation to directions of body movement and Japanese nori	38	Speaker (one Genelec 8050A)	Not reported

*The original information about the types of headphones or speakers given by the authors is shown in parentheses. The studies are listed in a chronological order*.

Besides the type and brand of equipment used, the playback level is an important sound factor to consider when designing an embodied music cognition experiment (Todd and Cody, 2000). Table 1 therefore also includes the reported sound level (if any) in the selected studies. It turns out that several articles do not report the sound level at all, while others report it as “comfortable.” In the cases where measurements are provided, the sound levels are typically in the range of 60–90 dB. It should be mentioned, however, that measuring sound levels in an experimental setting is not straightforward. This is particularly true for headphones, for which a proper sound level measurement would involve a dummy head and calibrated microphones to get reliable results.

There are often pragmatic reasons for choosing a particular playback method over another for an experiment. In some cases, a laboratory is already equipped with a particular sound playback system. Other times, the experimental design may dictate the type of equipment to use. For example, while studying free movement to music—such as dancing—it is impractical to use wired headphones. Then, a speaker-based setup would be the most viable solution, although wireless headphones could also be considered. Also, some experimental rooms may have challenging acoustics and/or problems with leaking sounds to adjacent rooms. In such cases, headphones may provide a better overall setup for an experiment. While such reasons often legitimize the choice of a particular playback system, our small review shows that these choices are rarely described and discussed.

#### 1.1.3. Comparative Studies of Headphones and Speakers

Many of the previous studies on differences between headphones and speakers have been carried out in the fields of acoustics and sound engineering. In such studies, the focus is typically on the technical design of the equipment and the reproduced signal quality. We are more interested in the experiential differences between headphones and speakers, and thus, studies in, for example, speech science, are more relevant. One such study is that of Schmidt-Nielsen and Everett (1982), who found that mild fluctuations of pitch in synthetic vowels were more easily detected when the stimulus was presented using speakers instead of headphones. Another relevant field is traffic safety. In a study on the efficiency of simulated driving during music listening, Nelson and Nilsson (1990) showed that participants' reaction times for shifting gears were longer when using headphones than when using speakers. Interestingly, they also found that the subjective fatigue was the same in both conditions.

In a mixed-methods study, Kallinen and Ravaja (2007) compared the experience of listening to business news through headphones and speakers. Here, different types of physiological measures were collected: facial electromyography (EMG), pulse transit time (PTT), respiratory sinus arrhythmia (RSA), and electrodermal activity (EDA). They found that listening to news using headphones elicited more positive reactions (EMG activity of zygomaticus major) and higher attention (shorter PTT) compared with the use of speakers. Headphones listening was also preferred by most of the participants. However, while listening to speakers, participants who scored high on the sociability and activity personality scales showed increased attention (lower RSA), whereas impulsive, sensation-seeking participants showed higher physiological arousal (increased EDA). This study showed not only crucial differences in reception of speech from headphones and from speakers, but also that these differences can vary between people depending on their personality traits. Some years later, Lieberman et al. (2016) conducted a similar study, comparing the effects of headphones vs. speakers on how participants received emotional stories (personal confessions and requests for help). The authors found that listening to such stories through headphones increased the participants' feeling of the narrator's presence, their subjective immersion in the story, and their positive attitude toward the narrator, compared to when they listened to the same stories from speakers. Headphones listening also increased the participants' willingness to donate money. The authors conclude that listening to speech on headphones reduces felt social distance.

In the field of music perception, Koehl et al. (2011) investigated whether headphones can be used on equal terms with speakers in studies where listeners have to assess subtle differences between auditory sequences. In their study, expert listeners were asked to rate (by degree of similarity and personal preference) pairs of short baroque sonata excerpts while listening from headphones or from speakers. The stimuli had been recorded with two different microphone setups. The study revealed that the participants could distinguish the types of recordings equally well while listening to headphones or speakers, but the preference for one type of recording was slightly but significantly higher in the headphones condition. Furthermore, evaluating the excerpts through headphones resulted in greater consistency across participants. The authors attributed this difference to the fact that while listening to speakers, the participants could freely move their heads, which modifies the reception of sound. The headphones, on the other hand, were fastened on the participants' heads, which provided a stereo field independent of head movement. Despite these observed differences, Koehl et al. (2011) concluded that both playback methods are equally appropriate for studies in which listeners evaluate and rate musical excerpts.

Confirming common knowledge among audio engineers, King et al. (2013) showed that highly trained recording engineers and music producers worked differently while monitoring with either headphones or speakers. This was observed in how they set levels to balance solo musical elements against a backing track. The authors concluded that results from tests that used headphones as a playback method might not be generalized to situations where speakers are used, and vice versa.

One part of a music experience that differs significantly between headphones and speakers, is the perception of low frequencies. McMullin (2017) explored differences in loudness and bass level preferences while listening through the two types of devices. When asked to equalize sound parameters and adjust to a preferred sound level, the listeners set the loudness level 2 dB higher and the bass level 1 dB higher for the loudspeakers. Moreover, the variance in preferred bass and loudness levels was comparatively greater in the headphones condition. Interestingly, adjusting the bass level proved much more difficult with headphones than with loudspeakers. McMullin (2017) points out that listening on headphones deprives the person of whole body sensations of low frequency vibrations. This means that listeners have less tactile feedback to help them make decisions about the right bass level in music. Additionally, this study demonstrated that trained listeners were more consistent than untrained listeners in their bass level and sound volume level adjustments.

Another group of researchers discussed auditory experiments conducted remotely over the Internet, where researchers have little control over playback methods available to participants (Woods et al., 2017). They argued that headphones, which attenuate external noise and generally improve control over the basic quality of the presented stimulus, should be the preferred method of presenting sound. To verify the type of playback system participants are using, the researchers developed a short test of pure tones that are heard differently through headphones and speakers due to phase cancellation. While this study does not explicitly compare headphones and speakers, it supports the argument of treating them as unequal playback methods in experimental research.

To conclude, our review of studies comparing the experience of using headphones and speakers showed various differences between the two playback methods. Such studies typically focus on either speech or music perception. None of them, however, directly address bodily responses to music.

### 1.2. Body Movement as a Spontaneous Response to Music

There is a general belief that “music makes us move,” but the empirical evidence of such a claim is scarce. Many of the studies on music-related movement focus on voluntary and fairly large-scale displacements of the body (Gritten and King, 2006, 2011). When it comes to spontaneous responses to music, it is more relevant to consider the literature on postural sway (Forti et al., 2010; Ross et al., 2016; Coste et al., 2018) and subtle head nodding and tapping (Hurley et al., 2014; Kilchenmann and Senn, 2015).

#### 1.2.1. Music-Related Micromotion

Our main focus is on spontaneous, voluntary or involuntary movement of the body that occurs while experiencing music, what we call *micromotion*. We have studied micromotion using an experimental paradigm in which subjects are asked to stand still on the floor while listening to music (Jensenius et al., 2017). From these studies we have found that people's micromotion is on average higher when listening to music than when they stand still in silence, even when they deliberately try not to move (Jensenius et al., 2017; González Sánchez et al., 2018; González Sánchez et al., 2019). Different types of music seem to influence the micromotion in various ways. We have, for example, found that music with a clear pulse and rhythmic structure (such as found in electronic dance music, EDM) leads to higher levels of micromotion. This can be attributed to a number of factors—for instance, intensified breathing, body sway, or postural adjustments.

Our findings on micromotion are consistent with studies of physiological responses, suggesting that the experience of music can be reflected in various changes in human hormonal, cardiovascular, respiratory, thermoregulatory, muscular, and even digestive systems (Hodges, 2009). As Hodges points out, these physiological responses may also lead to physical responses to music in the form of body movement. Micromotion can also signify an ongoing rhythmic entrainment process (Large and Jones, 1999), which is demonstrated in periodic motion of the body synchronized to the beat of the music. A recent overview of studies concerning this phenomenon can be found in Levitin et al. (2018).

#### 1.2.2. Spontaneous Movement to Music

While our previous studies have been on music-related micromotion, the current experiment focused on slightly larger-scale movement. This could be in the form of head nodding or finger tapping, or other subtle body movement that spontaneously appears in response to music. Reviewing the literature, we see that some researchers use “spontaneous movement to music” to describe free, dance-like movement that participants are asked to perform (Luck et al., 2009; Toiviainen et al., 2010; Burger et al., 2013). Here, we focus on *spontaneously appearing* movement, that is, when participants are not instructed to move, or when they are instructed to move a different body part.

In an experiment on “attentive listening,” Kilchenmann and Senn (2015) investigated listeners' spontaneous body motion. They observed that participants spontaneously moved their heads to the beat of the music when they were asked to rate excerpts of swing and funk music with minute timing manipulations. This happened even though they were not given instructions to move, and they were not aware that their movements were being measured. Participants who identified as musicians reacted more strongly to the sonic manipulations, which was reflected in the intensity of their head movement. Hurley et al. (2014) took a different approach to measuring spontaneous body movement to music. They equipped participants with a drum pad and told them to tap to the music, if they wished. Apart from tapping data, they also recorded head motion, although no instructions about performing head movements were given to participants. Spontaneous motion synchronization to music was treated as a proxy for the participants' engagement, together with their ratings of the *groove* of the music, that is, the aspect of music that elicits an urge to move (Janata et al., 2012). The researchers found that music with “staggered” instrument entrances—that is, instruments entering one at a time, as opposed to simultaneously—elicited increased sensorimotor coupling. Furthermore, the musically trained participants were more eager to tap along with the music, and their timing was more accurate. However, the precision with which the participants synchronized their head movements to the music did not differ between the musically trained and untrained participants.

Another set of studies that have yielded interesting findings with regards to the effect of music on spontaneous body motion, is in the field of *posturography*. Here, postural control is studied when people stand upright in either static or dynamic conditions. In posturography studies the auditory stimuli are treated as external distractors that can affect the participants' balance. Ross et al. (2016) found that listening to music with high levels of groove reduced the radial body sway when standing. At the same time, it encouraged spontaneous motor entrainment to rhythmic events in the music without any instruction for such movement. Coste et al. (2018) demonstrated that discrete auditory rhythms can influence both voluntary and involuntary body sway, and induce movement entrainment to rhythm, especially when the rhythmic frequency is similar to the body's natural sway.

Although the correspondences between music and body movement are not yet fully understood, a number of theories have been proposed to explain why people often spontaneously start moving to music. One line of research highlights the existence of robust connections between the auditory and motor areas in the human brain (Zatorre et al., 2007), and the automatic activation of movement-related structures, such as the supplementary motor area, premotor cortex, and cerebellum, in response to auditory rhythms (Grahn and Rowe, 2009). This suggests that, at least to some extent, body movement can happen automatically, as a spontaneous, and perhaps even involuntary, response to music. Moreover, moving to music is universal among humans (Blacking, 1995), and people in all known cultures dance to music (Sievers et al., 2013). Indeed, many studies explain the phenomenon of moving to music from an evolutionary perspective, showing that the strong connections between sound and body movement are deeply rooted in human biology and culture (Levitin et al., 2018). Some researchers suggest that the synchronization of body movements to music was evolutionarily reinforced, because it promotes interpersonal cooperation and bonding (Reddish et al., 2013; Tarr et al., 2016). This may be a reason that moving to music—with or without other people—is strongly linked with pleasure (Solberg and Jensenius, 2017; Witek et al., 2017).

#### 1.2.3. The Effect of Musical Stimuli

While many musical features can potentially lead to spontaneous movement of the body, there is growing evidence that rhythmic elements may be particularly movement-inducing (Burger et al., 2013). That is probably the reason why many researchers tend to use music genres with clear rhythmic structures when studying music-related body movement. Several recent studies have focused on the genre of EDM (Moelants, 2003; Solberg and Jensenius, 2017; Burger and Toiviainen, 2018), which may also be seen as reflecting the uptake of this particular genre in a large part of today's popular music. Also in our own previous studies we have found that EDM makes people move more than other musical genres (Jensenius et al., 2017; González Sánchez et al., 2018).

There is, however, no consensus on how complex the music and its rhythmical structure should be in order to create the urge to move or to aid in movement synchronization. This topic has been explored in the context of rehabilitation of patients with diseases that affect their motor control (such as Parkinson's or Huntington's disease), but with no clear conclusions (Wittwer et al., 2013). Witek et al. (2014) argue that the rhythm should be neither too simple nor too complex. Styns et al. (2007) showed that people synchronize their walking cadence better with complex music than with simple rhythmic structures. It has also been suggested that the subjective enjoyment of a piece of music strongly influences the feeling of groove (Janata et al., 2012; Senn et al., 2018).

#### 1.2.4. The Effect of Individual Differences

In our previous studies we have found that time spent on physical exercise positively correlates with the amount of involuntary movement during standstill (Jensenius et al., 2017). We have also found a correlation with age, showing that younger people tend to move more than older people when trying to stand as still as possible (Jensenius et al., 2017; González Sánchez et al., 2018). Moreover, we observed positive correlations between body height and quantity of motion (González Sánchez et al., 2018). This is similar to results by Dahl et al. (2014), who found that the preferred tempo for dancing can be predicted by the height and leg length of the participants. This suggests that body morphology may influence the process of physically engaging with music.

As we have shown in sections 1.1.1 and 1.1.3, there are differences in preferred use for headphones and speakers. These differences might depend on the listening context, but also on the listener. For example, headphones are used more often by young adults than those who are above 45 years old (Fung et al., 2013). Having a broader understanding of the listeners' preferences and habits for using headphones and speakers could aid understanding their responses to music listened through these playback methods. To our knowledge, there are yet no studies on this topic.

#### 1.2.5. Movement Measures

Previous studies on body movement to music have investigated different body segments (Luck et al., 2010; Burger et al., 2013), movements of the head (Hurley et al., 2014; Kilchenmann and Senn, 2015; González Sánchez et al., 2018; González Sánchez et al., 2019), and Center of Mass (CoM) or Center of Pressure (CoP) (Burger et al., 2013; Ross et al., 2016). In posturography studies, CoM is one of the most widely used measures of postural stability (Winter, 2009). In music cognition research, however, there are no standard movement measures. In the present study, we analyzed three different measures: Head Motion (Head), Center of Mass (CoM), and Whole Body Motion (Body). The latter was calculated as an average of all markers (see below for details). We used these three measures in order to explore different kinds of movement responses to music. Additionally, each of these three movement measures is in a different way sensitive to postural adjustments and incidental fidgeting.

### 1.3. Research Questions and Hypotheses

In sum, there is some scientific evidence of differences between headphones and speakers in listening experience. There is also a growing body of literature on spontaneous body movement to music. However, to our knowledge, there has been no previous studies on the combination of these two topics: influence of playback method on spontaneous movement to music. Based on the above literature review, and our own previous findings, we therefore ask the following questions:

1. Will different playback methods (headphones and speakers) influence the quantity of observed spontaneous movement when people stand and listen to music?
2. Can any differences in observed movement be related to the musical complexity of the sound stimuli?
3. Can any differences in observed movement be related to the individual (demographics, musical preferences, and listening habits)?

Although previous knowledge is limited, we hypothesize that the playback method (headphones or speakers) will result in different spontaneous bodily reactions to the music. Given the exploratory nature of the study, and the lack of previous research on the topic, we do not have a prediction for the direction of the difference in movement. When it comes to the question of musical complexity, we hypothesize that a higher degree of musical complexity (with a particular focus on rhythmic complexity) will lead to a higher level of movement. The question of individual differences is exploratory, and we therefore do not have hypotheses for this question.

## 2. Method

The main objective of this study is to examine possible relationships between sound playback methods and spontaneous body movement. For that reason we designed a motion capture experiment in which participants listened to the same stimuli with both headphones and speakers. The study was constructed around a 2 × 2 × 3 ANOVA design: playback method × stimuli complexity × movement measure. In addition, we wanted to explore possible correlations between observed body movement and individual differences between participants. The study obtained ethical approval from the Norwegian Centre for Research Data (NSD), under the project identification number 58546.

### 2.1. Participants

A total of 42 participants were recruited to take part in the study via advertisements placed in several locations around the University of Oslo. The exclusion criteria included hearing loss, neurological disorders, arthritis, orthopedic conditions, recent injury, and balance disorders. A total of 5 participants were excluded from the analysis due to data loss, a misunderstanding of the instructions, and one late report of injury. Two more participants were excluded as outliers, because their quantity of motion exceeded 3 standard deviations (SD). Subsequently, 35 participants were included in the analyses (18 females and 17 males; average age: 27.1 years; SD: 5.4 years; average height: 176.3 cm; SD: 9.7 cm). The height was calculated as the mean value of each participant's vertical head position. Of the included participants, 24 reported that they had some musical training, either professional or self-taught, out of which 19 still regularly played an instrument or sang. All the participants were rewarded with a gift card worth NOK 200 (approximately EUR 20).

### 2.2. Music Stimuli

Based on findings from previous studies (Jensenius et al., 2017; González Sánchez et al., 2018; González Sánchez et al., 2019), we decided to focus on using EDM-like tracks in the present study. This is a musical genre that is designed specifically for making people want to dance, and is characterized by a flat-four rhythmic pattern and a synthesizer-based melody and accompaniment (Solberg and Jensenius, 2017). We believe that it is important to study the effects of “real” music, so four of the six selected tracks were taken from commercially available EDM tracks. Two custom-made control tracks were also included in the list of stimuli (the six tracks are described below, and details are provided in Table 2).

**Table 2:** An overview of the music stimuli used in the current study.

**Artist**	**Song title/Label/Year**	**Duration (s)**	**Tempo (BPM)**	**Event density**
—	Drum metronome	45	120	95
—	Two-measure drum pattern	45	120	115
André Bratten	Trommer og bass/Correspondant/2014	0:00–0:45	120	206
Neelix	Cherokee (Extended Mix)/Kontor Records/2017	4:32–5:17	138	253
Neelix	Cherokee (Extended Mix)/Kontor Records/2017	1:07–1:52	138	278
Pysh feat. Poludnice	Sadom (Original Mix)/Mono. Noise/2017	0:28–1:13	123	297

*The durations of the EDM stimuli refer to the extracted segments from the original tracks*.

The different tracks were selected because they have different levels of *musical complexity*. Musical complexity is here used to explain the combination of vertical and horizontal elements. The vertical elements include harmonic (combinations of individual tones), timbral (the sound of individual instruments), and textural (combinations of instruments) features. These vertical elements relate to the sonic “layers” of the music. EDM is to a large extent based on a combination of synthesized sounds and processed instrumental samples, and the different vertical layers often fuse into a complex texture in which it is not entirely straightforward to identify individual instruments or harmonic content (Brøvig-Hanssen and Danielsen, 2016). The horizontal (temporal) elements of EDM are based on its characteristic “flat-four” bass drum pattern, which drives the experience of a clear pulse of the music. On top of such a bass pattern there are often various layers of micro-rhythmic structures, as well as melodic lines. While seemingly simple in structure, the final “sound” of an EDM track is often composed of a large number of horizontal and vertical layers. It is characterized by a repetitive pattern, but often it is the micro-rhythmic *variation* that brings the music to life (Danielsen, 2010; Danielsen et al., 2019).

To reduce the number of independent variables, we decided to select EDM tracks that would allow for comparing the *rhythmic complexity* between stimuli in a systematic manner. By rhythmic complexity we here refer to the number of elements contributing to the rhythmic structure. All of the chosen stimuli have a clear pulse, but they have an increasing number of rhythmic elements that contribute to the overall rhythmic complexity. For example, a plain metronome can be considered to have a low level of rhythmic complexity, while an elaborate EDM track will have a high level of rhythmic complexity. Only tracks without lyrics were selected, to focus on the non-verbal content of the music. The six selected stimuli were:

1. *Metronome*: A plain metronome track based on a synthesized “EDM-style” drum sample.
2. *Rhythm*: A simple two-measure drum pattern adapted from the study by Honing et al. (2012). This was produced using the same synthesized drum sample as used in the metronome track.
3. *Bratten*: An excerpt from the beginning of the song *Trommer og bass* by André Bratten. This was chosen as an example of a professionally produced EDM track with a low level of complexity. It consists of a basic, steady rhythm, and no melody. Thus, it resembles the Rhythm track, but with richer and more interesting sonic qualities.
4. *Neelix1*: This is an excerpt from the trance track *Cherokee* by Neelix. It contains a complex rhythmic structure, including micro-rhythmic features, as well as several layers of bass and melody lines.
5. *Neelix2*: This is an excerpt from a different part of the same track as Neelix1. The main difference is that this track contains a small “break routine,” with a build-up of rhythmic layers and an upwards moving glissando.
6. *Pysh*: This is an excerpt from the deep house track *Sadom* by Pysh. It is based on a steady, but slightly laid-back beat, consisting of samples of acoustic drums. The use of a sampled voice (but no lyrics) also makes it perceptually different from the other tracks.

**Figure 1:**
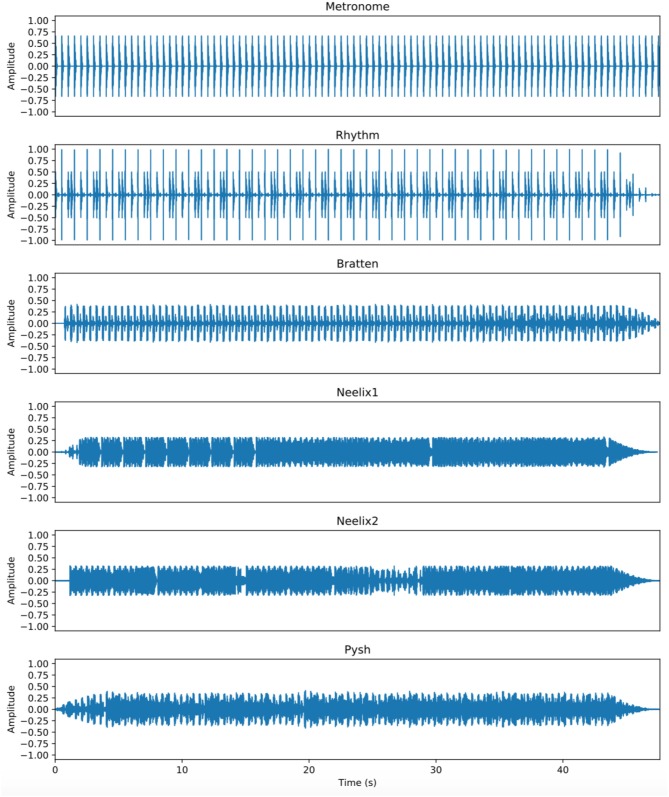
Waveform displays of the six sound stimuli show the increasing level of musical complexity from top to bottom (Metronome = low complexity, Pysh = high complexity). The amplitude peaks of the Metronome and Rhythm tracks are higher than those of the four EDM tracks, but they contain much less energy between the beats. The tracks were judged to be perceptually similar in loudness (see text for details).

**Figure 2:**
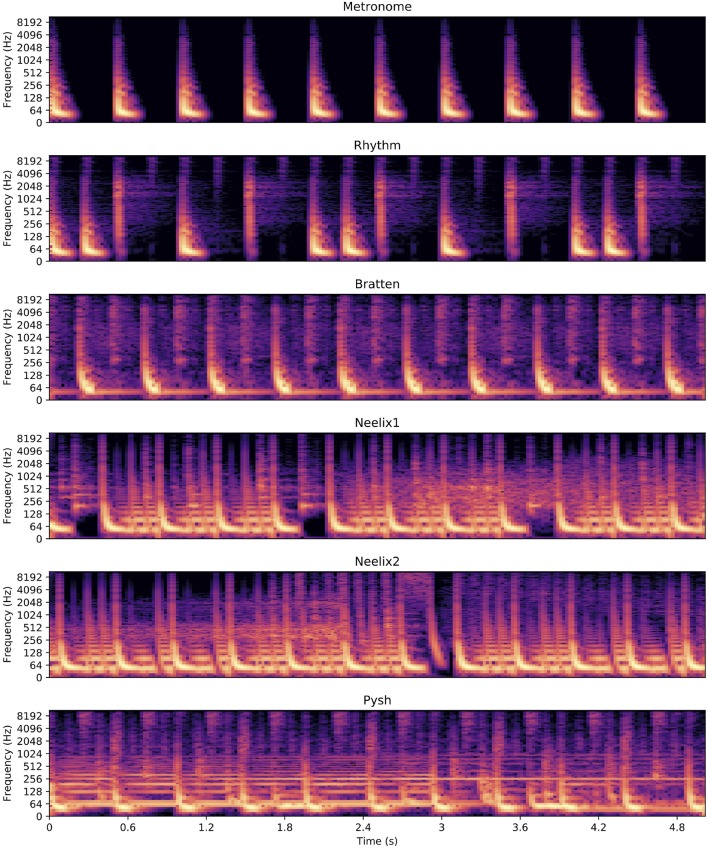
Spectrograms of 5 s of each of the six sound stimuli. These close-ups reveal the differences in musical complexity between the different tracks: low for the metronome (top) and higher for the EDM tracks (bottom).

To summarize, three of the tracks were primarily rhythmic in nature (Metronome, Rhythm, Bratten), yet with an increasing level of rhythmic complexity. The three other tracks had even more rhythmic complexity, but also contained more melodic layers. The increased complexity can be seen in the amplitude plots (Figure 1) and spectrograms and (Figure 2) of the sound files. Each track was ~45 s in duration (cut to match the bars), with small fade-ins and fade-outs for the excerpts that were cut from original EDM tracks. All the stimuli were in quadruple meter, contained no lyrics, and the tempo varied from 120 to 138 BPM. The tracks were created/modified in the Reaper digital audio workstation.

During the experiment, each of the six music stimuli were played in random order. The sound tracks alternated with 30-s segments of silence, and there were also silence segments in the beginning and end of the experiment. The total duration of the experiment was ~8 min. Since the tracks differed so much in their musical content, it was not possible to do a signal-based normalization of the loudness level. Therefore, the loudness level of each track was adjusted by ear by three of the authors during the pilot phase. This was done by listening to pairs of tracks, and adjusting the levels of each pair until the three listeners agreed that the perceptual sound level of the tracks was similar. The same procedure was used to adjust the levels between speakers and headphones. That is, three of the authors listened to each track with both playback methods, and adjusted the levels until they matched perceptually. The consistency of the perceived loudness level between playback methods and between tracks was validated by the participants of a pilot study conducted prior to the experiment.

### 2.3. Apparatus

**Figure 3:**
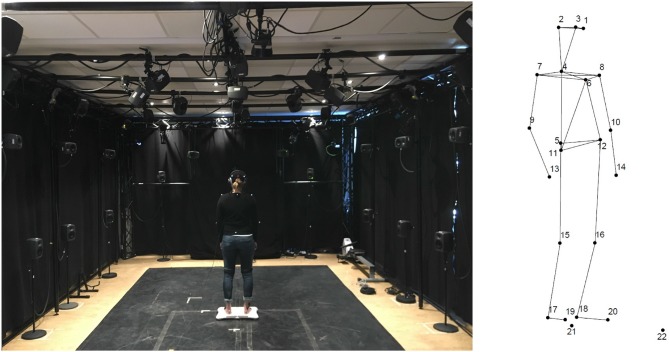
**(Left)** Laboratory setup and one participant standing during the headphones condition. Written informed consent was obtained from the participant for publication of images. **(Right)** Motion capture reconstructed markers and segments. The markers were located as follows (L, left; R, right; F, front; B, back): 1—F head; 2—RB head; 3—LB head; 4—B neck; 5—sacrum; 6—sternum; 7—R shoulder; 8—L shoulder; 9—R elbow; 10—L elbow; 11—R hip; 12—L hip; 13—R wrist; 14—L wrist; 15—R knee; 16—L knee; 17—R heel; 18—L heel; 19—R toe; 20—L toe; 21—reference marker on the Wii board; 22—reference marker on the floor.

The motion capture data collection was done using 20 reflective markers attached to relevant anatomical landmarks on the body of the participants (Figure 3). An infrared optical marker-based motion capture system from Qualisys (12 Oqus cameras) was used in the study, running at a 200 Hz sampling rate. The data was recorded and pre-processed in Qualisys Track Manager, and exported as TSV files for further analysis.

The sound stimuli were played from a laptop running a custom-built patch developed in Max by Cycling '74. This patch ran the stimuli in randomized order, and was also set up to synchronize with the motion capture system. All the sound stimuli were played from uncompressed audio files (.WAV), using an RME MADIface Pro sound card. The headphones used in the experiment were a pair of Beyerdynamic DT 770 PRO 80 Ohm; they were carefully placed on the participant's head and the headband was adjusted for their comfort. The speakers were a pair of Genelec 8020 loudspeakers with a Genelec 7050 subwoofer. The speakers were placed in a triangle configuration, each at a distance of 315 cm from the participant. They were mounted on a stand at a height of 165 cm, and with a distance of 290 cm between speakers. The subwoofer was placed on the floor equidistant between the speakers, and 245 cm away from the participant. The sound level of both playback systems was set to a level that was loud, but not uncomfortable. The sound level was set to 72 dB for the speakers and 74 dB for headphones. The difference was based on the perceptual matching done prior to the experiment (see above). The difference in 2 dB was also applied in McMullin (2017), to compensate for a lack of cross-talk in headphones condition. To determine that the sound level was indeed loud but not uncomfortable, a short sound check was done prior to the headphones session. A total of eight participants asked for lowering the sound level (to either 72 or 68 dB).

### 2.4. Questionnaire Measures

The participants were asked to fill in a short questionnaire after each of the two listening sessions (headphones and speakers). These included questions about felt movement, tiredness, and the perceived loudness (Table 3). At the end of the experiment, the participants filled in a longer questionnaire on demographics and listening habits (such as frequency of using headphones and speakers, see Table 4), and a Short Test of Music Preferences (STOMP; Rentfrow and Gosling, 2003). They were also presented with short excerpts of the music stimuli, and asked to evaluate how much they liked listening to them during the experiment. Three additional questionnaires, which are not a part of the current analysis, were filled in between the listening sessions and as a part of the final questionnaire.

**Table 3:** Mean, standard deviation, and median values for the answers to questions asked after each listening session: “Did you feel that you were moving?”; “Did you feel tired during standing?”; “Did you perceive the music as loud?” (*N* = 35).

	**Moving**			**Loudness**			**Tiredness**		
	**Mean**	**SD**	**Median**	**Mean**	**SD**	**Median**	**Mean**	**SD**	**Median**
Headphones	2.7	1.1	2.0	2.4	1.2	2.0	2.5	1.1	3.0
Speakers	2.7	1.1	3.0	2.3	1.1	2.0	2.2	1.1	2.0

*The answers were provided on a 5-point scale ranging from “No” to “Very much” (no descriptions in between; coded as ranging from 1 to 5)*.

**Table 4:** Questions about headphones and speakers—experiences during the experiment and habits of using both playback methods in everyday life (*N* = 35).

**Question**		**Mean**	**SD**	**Median**
1	Which part of the experiment felt more comfortable—headphones or speakers?	3.3	1.2	3.0
2	Did you feel that you moved more while using headphones or speakers?	3.0	1.1	3.0
3	Did you perceive music in headphones or in speakers as louder?	2.6	1.3	3.0
4	Do you enjoy listening to music at loud volume from headphones?	3.3	1.3	4.0
5	Do you enjoy listening to music at loud volume from speakers?	3.5	1.3	4.0
6	How often do you use headphones to listen to music?	58%	29%	60%
7	How often do you use speakers to listen to music?	42%	29%	40%

*For questions 1–5, answers were provided on a 5-point scale: for questions 1–3, ranging from “Definitely headphones” to “Definitely speakers,” and for questions 4 and 5, ranging from “Definitely not” to “Very much” (no descriptions in between; coded as ranging from 1 to 5). For questions 6 and 7, the answers were formulated as the “_% of the time”*.

### 2.5. Procedure

The experiment took place in the fourMs Lab at the University of Oslo between April and May 2018. The participants were invited to the laboratory individually and were asked to give written consent before the study began. Afterwards, the participants were instrumented for the first listening session, which was headphones listening for one half, and speakers listening for another half of the participants. Each group was presented with the same set of stimuli in a randomized order. Participants were randomly assigned to one of the groups (starting with headphones or starting with speakers). In the final sample, 11 females started with headphones and 7 with speakers, and 7 males started with headphones and 11 with speakers. Participants were asked to put on a motion capture suit, and EMG electrodes were placed on each foot, forearm and shoulder. In addition, a breathing sensor was placed on the torso. The EMG and respiration measurements were added for methodological experimentation, and will not be included in the current analysis. The same is the case for the data from the Wii balance platform that the participants were standing on (see Figure 3 for illustration of the setup in the lab).

When ready, participants were asked to stand on the balance platform and remain in a relaxed, comfortable position during the experiment. They were asked to look in the direction of a white cross placed on a black wall in front of them (340 cm away from the platform). No specific instructions to move to the music or to try to stand still were provided (see Appendix for a script of the instruction). After the first recording session, the participants were asked to sit down and fill in the first set of questionnaires. When the participants were ready, the second listening session took place, followed by the filling in of the remaining set of questionnaires. The total duration of the experiment was around 1 h 15 min, with small variations depending on time spent on preparation and on filling in the questionnaires.

### 2.6. Analysis

Analysis of the motion capture data started with the extraction of the position of the Center of Mass (CoM) based on the position of the marker placed on the sacrum (lower back) as in Mapelli et al. (2014). Next, head position was calculated from the middle point between the markers placed on both sides of the parietal area of the head (see Figure 3 for reference). The Whole Body Motion (Body) was measured by calculating the average position of all 20 markers for each sample. Head position data from two participants were incomplete, and therefore, were excluded, resulting in a sample of 33 participants for the head position data and a sample of 35 participants for CoM and Body data. The magnitudes of the CoM, Head, and Body velocity vectors were computed by differentiating the position data. The extraction of position data and computation of velocities were done in Matlab using the MoCap toolbox (Burger and Toiviainen, 2013). Mean CoM, Head, and Body velocity data for each participant and each session were then split into music and silence segments, and only the music segments were used for the statistical analysis. Although initially an analysis of silence segments, and a comparison of silence and music segments, were planned, they were not performed due to procedural problems that are described in the Discussion.

Analysis of the sound stimuli was performed using the MIRtoolbox (Lartillot et al., 2008). We decided to focus on rhythmic complexity, and this was measured based on the *event density* of the tracks. This feature was extracted with the mireventdensity function of the MIR Toolbox, and is based on counting the peaks of the envelope of the waveform. The event densities are summarized in Table 2. The median value of the six tracks was 229.5 events, and this value was used to separate the stimuli into two categories: low (Metronome, Rhythm, Bratten) and high (Neelix1, Neelix2, and Pysh) event density.

Analysis of the questionnaire and movement velocity data was performed using IBM SPSS Statistics 25. A repeated measures 2 × 2 × 3 ANOVA was performed with playback method (headphones/speakers), event density (low/high), and movement measure (Head/CoM/Body) as within-subject factors, in order to assess the significance of each factor and potential interactions between factors. Due to the non-normal distribution of scores in some of the questionnaire items, Spearman's rank correlations were computed between questionnaire and movement data, as well as between relevant questionnaire items.

## 3. Results

### 3.1. Motion Capture Data

The results of the 2 × 2 × 3 repeated measures ANOVA showed a significant effect of the playback method [*F*_(1, 32)_ = 10.09, *p* = 0.003] and a significant effect of the movement measure [*F*_(1, 64)_ = 74.48, *p* < 0.001, with a Greenhouse-Geisser correction] on the observed movement velocity, as well as an interaction between the playback method and the movement measure [*F*_(1, 64)_ = 6.61, *p* = 0.013, with a Greenhouse-Geisser correction]. No significant effect of the event density, and no interaction between the event density and playback method or between the event density and movement measure were observed.

To explore the interaction between the playback method and movement measure, we performed repeated measures ANOVAs for each movement measure. These showed that the playback method had a significant effect on Head [*F*_(1, 32)_ = 9.07, *p* = 0.005] and Body [*F*_(1, 34)_ = 4.61, *p* = 0.039], but it did not have a significant effect on CoM [*F*_(1, 34)_ = 2.43, *p* = 0.129].

**Figure 4:**
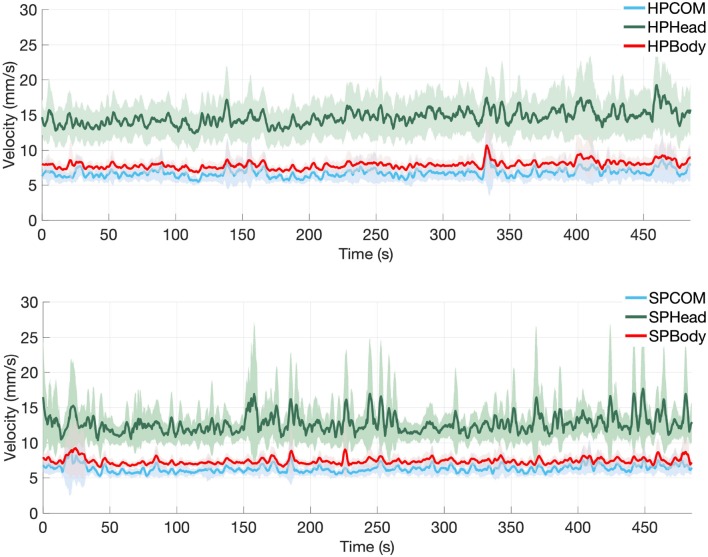
Mean velocity values of Head Motion (Head), Center of Mass (CoM), and Whole Body Motion (Body) across all participants, with 95% confidence intervals for the headphones condition (top) and for the speakers condition (bottom) for the whole trial (a total of 480 s, consisting of alternating segments of 30 s of silence and 45 s of sound stimuli, starting and ending with silence).

Means and standard deviations of the Head, CoM and Body velocities of all participants in both the headphones and speakers conditions are shown in Table 5. Figure 4 shows the means and confidence intervals for all participants across the whole 8-min session.

**Table 5:** Values of the mean and standard deviation of the velocity (mm/s) for each of the motion measures (Head, Center of Mass, Body) during music listening in each condition.

	**Head (*****N*** **=** **33)**		**CoM (*****N*** **=** **35)**		**Body (*****N*** **=** **35)**	
	**M**	**SD**	**M**	**SD**	**M**	**SD**
Headphones	14.4	6.8	6.6	1.8	7.8	1.8
Speakers	11.7	3.2	6.2	1.6	7.3	1.3

*The discrepancy in included participants is due to missing head markers data from two participants*.

No significant movement velocity differences were observed between male and female participants in any of the three movement measures, when compared using an independent samples *t*-test. However, a significant correlation was found between the participants' height and their Head data in both headphones (*rp* = 0.393, *p* = 0.024) and speakers (*rp* = 0.440, *p* = 0.01) conditions. This indicates that taller participants on average moved their head more during music listening. No significant correlations were found between the participants' height and their Body or CoM data in the two listening conditions.

### 3.2. Questionnaire Data

A Wilcoxon test was performed for the questions that were answered after each listening session. The analysis showed that participants reported feeling more tired during the headphones listening session (Mdn = 3) than during speakers listening session (Mdn = 2) (*Z* = −2.049, *p* = 0.040). No significant differences in perceived loudness or perceived amount of movement were observed (Table 3).

Means and standard deviations of answers to further questions that related directly to using headphones and speakers, either in everyday life or during this experiment, are reported in Table 4. With regards to the question, “Which type of headphones do you usually use?” usage of in-ear headphones was reported 19 times, on-ear headphones 11 times, and around-ear headphones 12 times. A schematic picture of each type of headphones was included in the questionnaire, to ensure that the participants understood the question. Two participants reported not using headphones at all in their everyday life (6% of participants), 25 reported using one type (71% of participants), 7 using two types (20% of participants), and one using all three listed types of headphones (3% of participants).

A Spearman's rank correlation revealed that enjoyment of listening to music played loud on headphones correlated with the regularity of headphones use (*rs* = 0.364, *p* = 0.032) and enjoyment of listening to music played loud on speakers (*rs* = 0.563, *p* < 0.001). A positive correlation was found between enjoyment of listening to music played loud on headphones and hours spent weekly listening to music (*rs* = 0.339, *p* = 0.047), and, at a trend level, between regularity of speakers use and age (*rs* = 0.332, *p* = 0.052).

Many significant correlations were found between habits of using headphones and speakers, and enjoyment of the music stimuli used in the experiment. Enjoyment of listening to music played loud on headphones correlated with liking both songs by Neelix (*rs* = 0.641, *p* < 0.0001 and *rs* = 0.446, *p* < 0.0001) and an average liking of all stimuli (*rs* = 0.641, *p* = 0.007). It also correlated with liking the song by Pysh, but only at the verge of significance (*rs* = 0.332, *p* = 0.051). Enjoyment of listening to music played loud on speakers correlated with liking the song by André Bratten (*rs* = 0.471, *p* = 0.004), both songs by Neelix (*rs* = 0.348, *p* = 0.041 and *rs* = 0.361, *p* = 0.033), as well as with an average liking of all stimulus *rs* = 0.402, *p* = 0.017). Regularity of headphones use negatively correlated with enjoyment of the metronome track (*rs* = −0.358, *p* = 0.035).

The questions about headphones and speakers correlated with music preference scores from the STOMP questionnaire. These exploratory analyses revealed several significant correlations, which are reported in Table 6.

**Table 6:** Coefficients of Spearman's rank correlations between questions about headphones and speakers and STOMP items (*N* = 35).

**Question**	**Classical**	**Dance/Electronica**	**Religious**	**Pop**	**Heavy metal**	**Soundtracks/Theme songs**	**Reflective/Complex**	**Upbeat/Conventional**	**Energetic/Rhythmic**
1	0.251	0.103	−0.044	0.178	0.040	0.028	−0.153	−0.089	−0.023
2	0.454**	−0.045	0.233	0.035	−0.037	0.375*	0.055	0.348*	−0.185
3	−0.269	0.083	0.045	−0.046	−0.335*	−0.134	−0.217	−0.076	0.077
4	−0.175	0.678**	−0.112	0.245	0.330	−0.026	−0.188	−0.083	0.485**
5	0.193	0.225	−0.370*	0.023	0.527**	−0.106	−0.024	−0.195	0.182
6	−0.271	0.509**	0.019	0.366*	−0.101	0.218	−0.392*	0.107	0.282
7	0.271	−0.509**	−0.019	−0.366*	0.101	−0.218	0.392*	−0.107	−0.282

Significant values are marked in grey. **indicates significance at the 0.01 level and *at the 0.05 level. Questions numbers are explained in Table 4. No significant correlations were found for the following categories: Blues, Country, Folk, Rap/Hip-Hop, Soul/Funk, Alternative, Jazz, Rock, Intense/Rebellious.

### 3.3. Correlations Between the Questionnaires and Motion Capture Data

Spearman's rank correlations were performed between questionnaire data and the velocity of Head, CoM and Body. Here it was found that Head in the headphones condition correlated significantly with age (*rs* = −0.382, *p* = 0.028) and liking to dance (*rs* = 0.451, *p* = 0.008). Body velocity in the headphones condition correlated significantly with liking to dance (*rs* = 0.402, *p* = 0.017). The self-reported subjective feeling of moving more while listening to headphones correlated with the velocities of CoM (*rs* = 0.503, *p* = 0.002) and Body (*rs* = 0.392, *p* = 0.020) in the headphones condition. No significant correlations were found between the velocity measures and the responses to the STOMP questionnaire.

## 4. Discussion

We discuss below the results from the experiment with respect to the three research questions posed in the introduction: whether different playback methods influence the spontaneous movement (RQ1), and if so, whether these differences are related to the musical complexity of the stimuli (RQ2) and/or to the participants' individual differences (RQ3).

### 4.1. Movement Differences for Headphones and Speakers

The clearest finding from the present study is the significantly higher mean velocity of the Head and Body motion capture data during headphones listening as compared to speakers listening. There are several potential explanations for this finding. First, wearing headphones that cover the ears restricts the participants' capacity to hear ambient sounds of the environment. Previous studies have shown that wearing ear defenders increases postural sway in healthy subjects (Kanegaonkar et al., 2012). The similarity of ear defenders to the tightly fit around-ear headphones used in our study may lead us to extrapolate that this is a possible cause for the higher velocity of movement observed while listening to headphones. However, we have not found any studies that compare postural stability during headphones vs. speakers use, or between different headphone designs. If headphones (including different types of headphones) have a disruptive impact on balance, this playback method should perhaps be reconsidered in movement experiments, and especially in the field of posturography.

Another plausible explanation for why participants moved more while listening to music using headphones, is that they were able to better enjoy the music. Perhaps the proximate location of the sound from headphones results in stronger reactions to music due to the stimulation of the vestibular system, causing pleasurable sensations of self-motion (Todd and Cody, 2000; Todd et al., 2008). It could also be that the participants experienced the use of headphones as more natural or comfortable than listening to speakers. Similarly, if headphones do, indeed, increase the feeling of intimacy or safety, it is possible that they helped participants forget about the laboratory setting and the presence of the experimenter in the back of the room. There is, however, no direct evidence that people move more to music when they feel comfortable or safe, or when they listen attentively, but it seems likely that such factors are of importance.

Interestingly, the playback method did not have a significant effect on the CoM measurements. Moreover, Head data was notably higher than the data from both CoM and Body. These differences can be explained through the dynamics of balance and posture control, and the inverted pendulum model of human posture (Winter, 2009). In a stable, standing posture, CoM should always present a smaller range of motion when compared with distant body segments. Burger et al. (2013) showed how free movement to music differs significantly between body segments, and in particular between the head and the rest of the body. A clear pulse was shown to induce whole body movement, while percussiveness seemed to induce clearer patterns from the participants' heads and hands. Our data confirms that Head, CoM and Body should be treated as complementary measures that can to different degrees depict small spontaneous body movement during music listening. In the future, it would be worthwhile to explore which of these and similar measures (such as movement of particular limbs) are most effective for capturing body sway and posture adjustments, and which are best for analysing spontaneously occurring movements that synchronize to musical rhythms. Extracting various features of the stimuli, and correlating them with the qualitative and quantitative aspects of such movements, may help to understand which sound features are important for spontaneous movement responses to music.

Participants, on average, reported that listening to headphones during the experiment was more tiresome than listening to the speakers. This is an interesting finding, which to our knowledge has no precedent in comparative studies on headphones and speakers use. In a study by Nelson and Nilsson (1990), the participants who listened to music over headphones or speakers while driving in a car simulator did not report differences in fatigue. It should be noted, however, that in this study, music was used as a background for performing other tasks. The other comparative studies reported here did not include participant reports on general tiredness or listening fatigue. Also, to our knowledge, there have not been any studies on the relationship between headphones use and listeners' fatigue in different contexts. However, several authors claim that the pressure exerted by sound on the eardrums, together with the in-head localization of sound in headphones, commonly result in listening fatigue (Bauer, 1965; Iwanaga et al., 2002; Vickers, 2009).

### 4.2. Musical Complexity

The experiment was designed with using six stimuli with varying level of musical complexity. Our primary interest was on rhythmic complexity, although the tracks' complexity also varied in other musical dimensions. We decided to use event density as a measure of rhythmic complexity, and for grouping the stimuli into two categories (low and high complexity). This is, of course, a crude reduction of rhythmic complexity, but it still manages to capture some of the core differences between the tracks in an efficient manner.

We did not find a significant effect of the rhythmic complexity on the movement responses. While this may seem surprising, it is in line with the results from a different study using the same stimuli (González Sánchez et al., 2019). One explanation for the lack of significance here may be that it is primarily the presence of a steady beat that drives the spontaneous movement responses. This fits with findings from some of our previous studies, in which EDM has led to more movement than other types of music with less clearly defined beat patterns (Jensenius et al., 2017; González Sánchez et al., 2018). It could have been interesting to perform correlation analysis per track, and also to carry out a more detailed musical analysis of the tracks in question, but that was out of the scope for this article.

### 4.3. Individual Differences

As we discussed in the introduction, there are differences in how people like to use headphones and speakers. These differences can be partially explained by factors such as age and music preferences. We found that older participants use speakers more often than headphones. This is in line with the results of a survey reported by Fung et al. (2013), which showed that younger adults (age 18–44 years) listen to music on headphones more than older adults. Interestingly, Kallinen and Ravaja (2007) report that 60% of the participants expressed a preference for listening to the news on headphones, as opposed to 40% who preferred to use speakers. In our study, the average self-reported usage of both playback methods in everyday life turned out to be 58% for headphones and 42% for speakers. These results seem similar, but it is also important to consider that preferences and actual use are not equivalent. There are many possible reasons for why people would buy and use headphones or speakers in everyday life, even though they might prefer to use a different playback system (see section 1.1.1). Our data also shows that enjoying listening to loud music over headphones correlates with the regularity of using headphones, but no such analogous relationship was observed for speakers. This can be a relevant finding for studies that deal with listener preferences and styles of engaging with music, as they may be dependent on the playback method used in a given context.

Another interesting finding was that people who like dance music also like to listen to music at a loud sound level over headphones, and that they report to use headphones more often than speakers. While one might expect to encounter dance music played over speakers at parties, considering the current popularity of EDM (Watson, 2018), it is not surprising that people listen to it over headphones also during everyday activities. Listeners may turn up the sound level to boost the energizing effect of the music and increase the feeling of pleasure (Todd and Cody, 2000). However, a more thorough study on the personal use of music is needed to confirm such speculations. This could also shed light on whether people consciously use a specific playback method in order to obtain a specific feeling (or perhaps when listening to different genres), and not only for pragmatic purposes. When it comes to preference for music genres, our data show some interesting correlation patterns with playback method. For example, we find that a preference for heavy metal music correlates with liking of listening to music played loudly on speakers, but not on headphones. This finding could aid the design of a study that focuses on this genre or includes such music material.

### 4.4. Limitations

There are several limitations in the design of this study. One is possible familiarity effects, since all participants had to listen to each stimulus twice (using both headphones and speakers). People generally tend to like songs that they have heard before more than when they listen to them for the first time (Peretz et al., 1998), even though after a certain number of repetitions, the positive affect becomes diminished (Hargreaves, 1984). Such changes in affect could be reflected in bodily responses to music; the data from the second session may be different from the first one simply based on the fact that the participants were already familiar with the stimuli. However, since the presentation order was counterbalanced between participants, we believe that it should not be considered as a bias factor for this study.

Another limitation of this study is that only one type of headphones and speakers were used. The choice of around-ears headphones and a stereo speaker setup was motivated by the common occurrence of these two scenarios in research on music-related body movement. As mentioned earlier, there are many different types and brands of headphones and numerous speaker configuration possibilities. It would be interesting to conduct more studies that investigate in more detail the effects of both different types and designs of the playback devices.

The decision to only include EDM-like music stimuli in this article may also be considered a limitation. Even though we found that EDM music has a particularly strong effect on body movement in our previous studies (Jensenius et al., 2017; González Sánchez et al., 2018), using other types of music (e.g., classical) may have produced different results. Therefore, the findings of this study should not be generalized to all genres of music. It could also be mentioned as a limitation that we were using real music with a lot of different musical variables. This was the reason we decided to include the two “synthetic” control tracks (Metronome and Rhythm) alongside the real music examples. In the future it would be interesting to try to get access to a real-world multi-track recording. Then it would be possible to experiment with the different musical layers in a more systematic manner.

We made sure that the participants included in this study had not previously participated in any of our standstill studies, which have publicly been known as “Championship of Standstill” (Jensenius et al., 2017; González Sánchez et al., 2018; González Sánchez et al., 2019). This was because the experimental design here was slightly different than in the previous studies. In the present study, participants were not instructed to stand as still as possible; they were asked to stand on the platform in a relaxed, comfortable position, with their arms at the sides of their body, and to remain in this neutral position during the experiment (see Appendix for a script of the instructions). They were also instructed to look toward a white cross on the wall. However, these instructions, combined with prior knowledge of our previous studies, might have encouraged some participants to try to not move at all. At the same time, many participants felt free to move subtly to the rhythm of the music. Thus, participants may have interpreted the study instructions differently, leading to an increased between–participant variance in the motion capture data. Moreover, some participants seemed to treat the silence between the tracks as a break, using this time to discretely stretch, straighten their posture, etc. They might have thought that it was only their body movement in response to the music that would be analyzed. We were originally also interested in the movement during silence segments, but had to abandon this comparison due to these inconsistencies in the data. Fortunately, no instances of touching the headphones or adjusting the motion capture suit were observed, neither during the music nor the silence segments. In future studies, more care should be taken when it comes to formulating the instructions in such a way as to avoid implicit directions to try not to move, and to ensure that the participants understand that the whole recording session, including silences, is to be analyzed.

Due to the nature of this study, it is not possible to conclude that the observed movement was fully dependent on the sound stimuli. However, by using three different motion capture measures, we aimed to reduce the probability of biases stemming from fidgeting and posture adjustments. For example, the CoM measure is less sensitive to arm movement. In the future, different types of movement analyses (for example, employing measures of entrainment to rhythm) might reveal further data about spontaneous body movement in both listening scenarios.

Individual differences, such as listening habits, music preferences, and body morphology, emerged as interesting factors in relation to spontaneous body movement in response to music listened over headphones and speakers. As indicated in the results of Kallinen and Ravaja (2007), some personal traits may influence body movements in response to music listened to through both playback methods. We believe that a more detailed analysis of individual differences could show interesting patterns in subtle, spontaneous body movement to music, also independently of the playback method.

## 5. Conclusions

Although there are still many open questions, this exploratory study has demonstrated that using headphones and speakers as playback methods can result in different patterns of body movement in a music listening experiment. Coming back to the original research questions, we can conclude that:

1. The participants moved on average more when listening to music with headphones than with speakers. This difference was particularly significant for head movement.
2. Complexity of the stimuli did not have a significant effect on the observed movement in headphones or speakers listening.
3. Individual differences correlate with body movement in response to music, and the pattern of these correlations is different for headphones and speakers listening.

Considering the potential effects of wearing headphones on postural control and the vestibular system, as well as the other features of both playback methods discussed in this article, careful choosing between them seems to be especially important for research paradigms in which the main interest is in body movement to music. Future studies are needed to better understand the impact of these two playback methods on bodily responses to music, and to explore potential differences between different types of headphones and speaker setups. Moreover, the patterns of preferences for music listening between headphones and speakers were shown to be asymmetrical, and the relationships between these preferences, listening habits, and individual traits should be further explored.

## Data Availability Statement

The datasets generated for this study are available on request to the corresponding author.

## Ethics Statement

The study was reviewed and approved by Norwegian Center for Research Data (NSD), under the project identification number 58546. The participants provided their written informed consent to participate in this study. Written informed consent was obtained from the individuals for the publication of any potentially identifiable images or data included in this article.

## Author Contributions

AZ, VG-S, AJ, and BL contributed to conception and design of the study. AZ and VG-S performed the experiments and statistical analysis. VG-S pre-processed the data. AZ wrote the first draft of the manuscript. AZ, VG-S, and AJ wrote the sections of the manuscript. All authors contributed to manuscript revision, read, and approved the submitted version.

## Conflict of Interest

The authors declare that the research was conducted in the absence of any commercial or financial relationships that could be construed as a potential conflict of interest.

## Appendix

The script used for the oral instruction given to participants at the beginning of the experiment:

Please stand on the force platform in a relaxed, comfortable position with your arms at the sides of your body. Try to remain in this neutral position during the experiment. Keep your eyes on the white cross on the wall. You will hear some rhythms and music, with periods of silence between them, and the experiment will last about 8 min. We will start with 30 seconds of silence. Is the instruction clear?
